# The Access Principle: The Case for Open Access to Research and Scholarship

**DOI:** 10.3201/eid1209.060643

**Published:** 2006-09

**Authors:** Marta Gwinn

**Affiliations:** * Centers for Disease Control and Prevention, Atlanta, Georgia, USA

**Keywords:** book review, The Access Principle, Marta Gwinn

Emerging Infectious Diseases helped pioneer open-access publishing by launching free online and print editions simultaneously in 1995. A decade later, perhaps half of the 50,000 scholarly journals are available online; however, access to the contents generally requires a subscription. Although many journals are experimenting with enhanced-access models, such as offering open access to a small selection of articles (e.g., Lancet) or making archived articles freely available 6–12 months after publication (e.g., New England Journal of Medicine), only ≈20% of all research articles are open access, and many of these are available only as self-archived manuscripts on authors' personal websites. Meanwhile, journal subscription rates continue to escalate, strapping library budgets and restricting circulation.

In this book, John Willinsky, professor of literacy and technology at the University of British Columbia, argues that access to the results of research and scholarship are a public good: information shared is not diminished; in fact, only when shared does it become knowledge. The access principle states that a commitment to research entails a responsibility to circulate the results as widely as possible.

Each chapter in the book presents this principle from a different perspective, making a case for open access on philosophical, ethical, practical, economic, and technical grounds. In each instance, the contentions of open-access critics are carefully dissected, exposed, and refuted with timely and relevant data. Individual researchers concerned about losing prestige and officers of professional societies concerned about losing subscription revenue might find these arguments particularly interesting. For example, the evidence from physics, a field with well-established open-access publishing conventions, suggests that open-access articles are cited more often (i.e., higher impact factor) than those available only to subscribers. Analyses of scholarly association budgets and journal management economics, which appear among the useful appendices at the end of the book, suggest that alternative publishing models could be more cost-effective than the status quo.

The author does not overlook the irony of publishing this work as a book that costs $34.95, but I tend to agree that the book is still the best medium for a "thoroughgoing treatment of an issue in a single sustained piece of writing." This book is for scholars and professionals who are interested in the idea of open access but are not yet convinced. Those who read it are likely to be surprised, engrossed, informed, and perhaps persuaded.

